# Evolutionary Patterns of Maternal Recognition of Pregnancy and Implantation in Eutherian Mammals

**DOI:** 10.3390/ani14142077

**Published:** 2024-07-16

**Authors:** Henrique Bartolomeu Braz, Rodrigo da Silva Nunes Barreto, Leandro Norberto da Silva-Júnior, Bianca de Oliveira Horvath-Pereira, Thamires Santos da Silva, Mônica Duarte da Silva, Francisco Acuña, Maria Angelica Miglino

**Affiliations:** 1 Ecology and Evolution Laboratory, Butantan Institute, São Paulo 05503-900, SP, Brazil; hb.braz@outlook.com; 2 Department of Animal Morphology and Physiology, Faculty of Agricultural and Veterinary Sciences, São Paulo State University, Jaboticabal 14884-900, SP, Brazil; rodrigo.barreto@unesp.br; 3 Department of Surgery, School of Veterinary Medicine and Animal Science, University of São Paulo, São Paulo 05508-270, SP, Brazil; leandronorberto@unimar.br (L.N.d.S.-J.); horvath@usp.br (B.d.O.H.-P.); thamiresssilva@usp.br (T.S.d.S.); monicasilva@usp.br (M.D.d.S.); 4 Department of Veterinary Medicine, University of Marília, Marília 17525-902, SP, Brazil; 5 Department of Chemistry and Biochemistry, The Ohio State University, Columbus, OH 43210, USA; 6 Facultad de Ciencias Veterinarias, Universidad Nacional de La Plata, La Plata B1900, Argentina; acunafranche@gmail.com

**Keywords:** pregnancy, evolution, implantation

## Abstract

**Simple Summary:**

For a pregnancy to begin, the embryo must “communicate” its presence to the mother’s body to prevent rejection. Also, the embryo must attach to the uterine lining (a process called implantation) to form the placenta, the organ that supports embryo growth. This review discusses how mammal embryos achieve these critical steps and how embryonic communication signals and implantation types have changed throughout mammalian history. Embryos release substances, such as proteins and hormones, to signal their presence to the mother’s body. These signals are species-specific, indicating that mammals have developed diverse mechanisms for pregnancy recognition. Implantation varies across mammal species. In most mammals, embryos attach to the surface of the uterine lining. This type of implantation has existed since the first mammals appeared on Earth and is widespread today, occurring in elephants, squirrels, horses, cows, whales, and many others. In some species, embryos partially or fully embed within the uterine wall. These implantation types have arisen many times in distantly related mammals, such as bats, rats, and great apes, including humans. Despite recent advancements, there is still much to discover. Future research should prioritize certain rodent and bat species to better understand how pregnancy recognition and implantation have evolved over time.

**Abstract:**

The implantation of the embryo into the maternal endometrium is a complex process associated with the evolution of viviparity and placentation in mammals. In this review, we provide an overview of maternal recognition of pregnancy signals and implantation modes in eutherians, focusing on their diverse mechanisms and evolutionary patterns. Different pregnancy recognition signals and implantation modes have evolved in eutherian mammals, reflecting the remarkable diversity of specializations in mammals following the evolution of viviparity. Superficial implantation is the ancestral implantation mode in Eutheria and its major clades. The other modes, secondary, partially, and primary interstitial implantation have each independently evolved multiple times in the evolutionary history of eutherians. Although significant progress has been made in understanding pregnancy recognition signals and implantation modes, there is still much to uncover. Rodents and chiropterans (especially Phyllostomidae) offer valuable opportunities for studying the transitions among implantation modes, but data is still scarce for these diverse orders. Further research should focus on unstudied taxa so we can establish robust patterns of evolutionary changes in pregnancy recognition signaling and implantation modes.

## 1. Introduction

Viviparity has independently evolved more than 150 times in vertebrates, characterizing one of the most remarkable examples of convergent evolution [1]. Despite this frequent evolution, viviparity requires many complex phenotypic changes to allow the internal development of embryos [2]. A fundamental change is the establishment of intimate contact between the embryo and maternal tissue, known as placentation. In mammals, maternal recognition of pregnancy and blastocyst/conceptus implantation are crucial for establishing pregnancy and ensuring successful placental development. 

Implantation is the process by which the blastocyst establishes physical and physiological contact with the maternal endometrium [3]. It is an intricately controlled process that depends heavily on the precise coordination of biochemical and molecular signals between the mother and the developing embryo [3,4,5]. In addition, mother-fetus communication involves mechanical and immunological interactions. Implantation may only occur during a receptive period of the maternal endometrium, known as the window of receptivity, initiated by the maternal recognition of pregnancy. In eutherian mammals, maternal recognition of pregnancy refers to the physiological process in which the embryo signals its presence to the maternal organism, thus preventing the activation of mechanisms that cause luteolysis [6]. Many embryonic losses in mammals are attributed to failures during maternal recognition of pregnancy and implantation [7,8]. Consequently, much effort has been placed into understanding these processes. However, less emphasis has been placed on the evolutionary patterns of these two phenomena.

Here, we provide an overview of the diversity of mechanisms and the patterns of evolutionary changes in maternal recognition of pregnancy and implantation in eutherians. We highlight commonalities and divergences of mechanisms among species and discuss the evolution of maternal recognition of pregnancy and implantation. We also suggest future studies on certain target lineages that will contribute to a better understanding of the evolution of these phenomena.

## 2. Preimplantation Development

Preimplantation development lays the foundation for establishing a successful pregnancy. It involves complex molecular and cellular processes that are mostly conserved across mammalian species. The general pattern of preimplantation embryonic development is common to all mammal species. The preimplantation phase begins with fertilization in the oviduct, where the single-celled zygote undergoes repeated and timely mitotic cell divisions (cleavage) to form a multi-celled, hollow structure termed the blastocyst [9]. The blastocyst is encapsulated within the zona pellucida and consists of an inner cell mass (the embryoblast), which will form the embryo, and an outer layer of cells (the trophoblast), which will form the embryonic part of the placenta (chorion). The trophoblast layer surrounds a fluid-filled cavity (blastocoel). In the uterus, the blastocyst hatches from the zona pellucida, likely as a combination of mechanical forces from blastocyst expansion and proteolytic activity by the trophectoderm cells [10]. Hatching renders the blastocyst competent for implantation by exposing the outer surface of the trophectoderm to the uterine environment.

The timing of blastocyst formation is generally conserved across mammals, typically occurring from 4–7 days after fertilization in most species [11,12,13]. However, the duration of the preimplantation phase varies considerably across eutherian mammals [12,14]. One factor contributing to this variation is embryonic diapause, a temporary suspension of embryonic development at the blastocyst stage that results in delayed implantation into the endometrium [15]. Embryonic diapause has been observed in at least 130 mammal species, including approximately 37 marsupials (Diprotodontia) and 94 eutherians (Carnivora, Rodentia, Eulipotyphla, Chiroptera, Xenarthra, and Artiodactyla) [16]. The occurrence of embryonic diapause in such a wide range of taxa, as well as the diversity of regulatory mechanisms, suggests that this strategy evolved independently multiple times in therian mammals in different habitats and under varying selective pressures [15,17,18]. Alternatively, embryonic diapause may have evolved a few times or just once in the therian ancestor, and its regulatory mechanisms have evolved to exploit it in advantageous species-specific contexts [18]. This assumption is based on the widespread occurrence of facultative diapause, the ability to induce diapause in non-diapausing species, the restriction of diapause to the blastocyst stage, and the intrageneric variation regarding the presence of diapause [18]. Embryonic diapause enables delayed implantation, providing adaptive advantages in challenging environmental conditions.

Another factor that contributes to the variation in the duration of preimplantation is the extent of blastocyst development after hatching. For example, in murid rodents and hominid primates, the hatched blastocyst remains small and spherical, and it implants in the uterus shortly after hatching. In murid rodents, this occurs within 6 h [19], whereas in humans it takes 1–2 days [20]. In contrast, in perissodactyls (e.g., horses) and certain artiodactyls (e.g., bovids, camelids, suids, and antilocaprids), the hatched blastocyst elongates extensively within the uterine lumen, and implantation initiates later, within 7–11 days after hatching [21,22,23,24]. One important implication of this divergence is that implantation precedes the development of extraembryonic membranes in rodents and primates, whereas it follows the development of extraembryonic membranes in artiodactyls and perissodactyls. At the onset of implantation, the yolk sac in bovids is partially formed [25], and the allantois begins to differentiate [26]. Blastocyst elongation has been identified in equids, bovids, suids, camelids, and antilocaprids, which suggests that this trait is an evolutionary novelty of the common ancestor of Perissodactyla + Artiodactyla. Overall, the duration of the preimplantation phase remains unknown in most mammals, and therefore, further research is needed to comprehensively understand and reconstruct the evolution of this trait.

## 3. Maternal Recognition of Pregnancy

Upon arrival in the uterus, the blastocyst undergoes a series of coordinated interactions with the uterine epithelium to ensure successful attachment and subsequent development. During this process, the blastocyst initiates signaling for pregnancy recognition [3]. Embryo signaling for maternal recognition of pregnancy has been essentially identified in livestock, domestic, and laboratory animals, as well as in humans [4]. Across all mammals (except carnivores), maternal recognition of pregnancy involves an embryonic signal aimed at preventing luteolysis, although the nature of this sign varies. Embryo signaling can have three primary effects on the corpus luteum: luteotrophic (promoting luteal function), antiluteolytic (preventing the luteolytic prostaglandin F_2α_ from reaching the corpus luteum), and luteostatic (protecting the corpus luteum from the luteolytic action of prostaglandin F_2α_). However, pregnancy recognition signaling varies widely across species and involves various embryonic factors, including glycoproteins, cytokines, steroids, and peptide hormones.

Estradiol (E_2_) mediates maternal recognition of pregnancy in the suid *Sus scrofa* [27,28]. E_2_ interacts with estrogen receptors in the endometrium, activating a mechanism that redirects the secretion of prostaglandin (PG) F_2α_ from the uterine vasculature to the endometrial lumen [29]. In the uterine lumen, PGF_2α_ is sequestered and metabolized to prevent luteolysis. E_2_ has also been proposed as the embryonic signal for pregnancy recognition in another Suina, the tayassuid *Pecari tajacu* [30], as well as in a Tylopoda, the camelid *Lama glama* [31,32]. E_2_, along with a placental luteotropin, appears to mediate maternal recognition of pregnancy in the rabbit *Oryctolagus cuniculus* [4,33]. Two hormone expression events mediate maternal recognition of pregnancy in rodents, specifically the rat (*Rattus norvegicus*), the mouse (*Mus musculus*), and the golden hamster (*Mesocricetus auratus*). Firstly, pituitary prolactin is secreted in semicircadian surges in response to penile stimulation of the uterine cervix during coitus. From day 12 of pregnancy until term, lactogenic hormones replace prolactin [34]. A placental lactogen secreted by an attached blastocyst has been proposed as the embryonic signal in elephants [35,36]. Chorionic gonadotropin (CG), a glycoprotein hormone, is the embryonic signal in anthropoid primates. CG molecules act as a luteotrophic factor, binding to luteinizing hormone/CG receptors on the corpus luteum, thus preventing luteolysis [37,38,39]. The cytokine interferon-τ (IFN-τ) is the embryonic signal in pecoran ruminants (sheep, cows, and goats) [29]. IFN-τ acts on the endometrium, inhibiting the PGF_2α_ synthesis [29,40]. 

Maternal recognition of pregnancy has been extensively studied in horses, but it remains unresolved. The equine conceptus migrates between uterine horns until attachment to the endometrium occurs, triggering an antiluteolytic mechanism that reduces PGF levels in the uterus and uterine venous plasma. Although the precise nature of the pregnancy recognition signal in horses is unknown, it is hypothesized to result from a physical rather than biochemical interaction between the embryo and endometrium [23], as well as a combination of mechanical and an unidentified chemical signal, mediated by a complex interplay of multiple signaling pathways [41]. Certain carnivores (e.g., cats, minks, dogs, and ferrets) lack classical maternal recognition of pregnancy [33,42]. These species lack embryonic signaling, and the corpus luteum persists for approximately the same duration in both pregnancy and nonpregnancy cycles [11]. In dogs, a broader definition of maternal recognition of pregnancy has been advocated, defining it as a morphological and functional relationship between the uterus, embryo, and corpus luteum [42]. 

In marsupials, maternal recognition of pregnancy has been recognized in macropodids and potoroids, based on conceptus-induced endometrial proliferation [43,44,45]. Maternal recognition of pregnancy has been hypothesized to occur in the dasyurid *Sminthopsis macroura* [44,46], but this assumption has been argued to lack robust evidence [47]. Macropodidae and Potoroidae are sister groups belonging to Diprotodontia, and therefore, maternal recognition of pregnancy was considered to have evolved in this clade [48]. Recent research has revealed that the presence of an embryo elicits transcriptional, protein, and morphological responses in the endometrium of the didelphid *Monodelphis domestica* (Didelphimorphia) [47]. These results suggest that at least some degree of pregnancy recognition, i.e., local endometrial recognition of pregnancy, is widespread in therian mammals [47]. However, the signaling mechanism in these marsupials is unknown. A chorionic gonadotrophin, detected in the early placenta of the tammar wallaby (*Notamacropus eugenii*), has been suggested as a potential hormone candidate, but no further study has been conducted to test this hypothesis [43,45].

**Figure 1:**
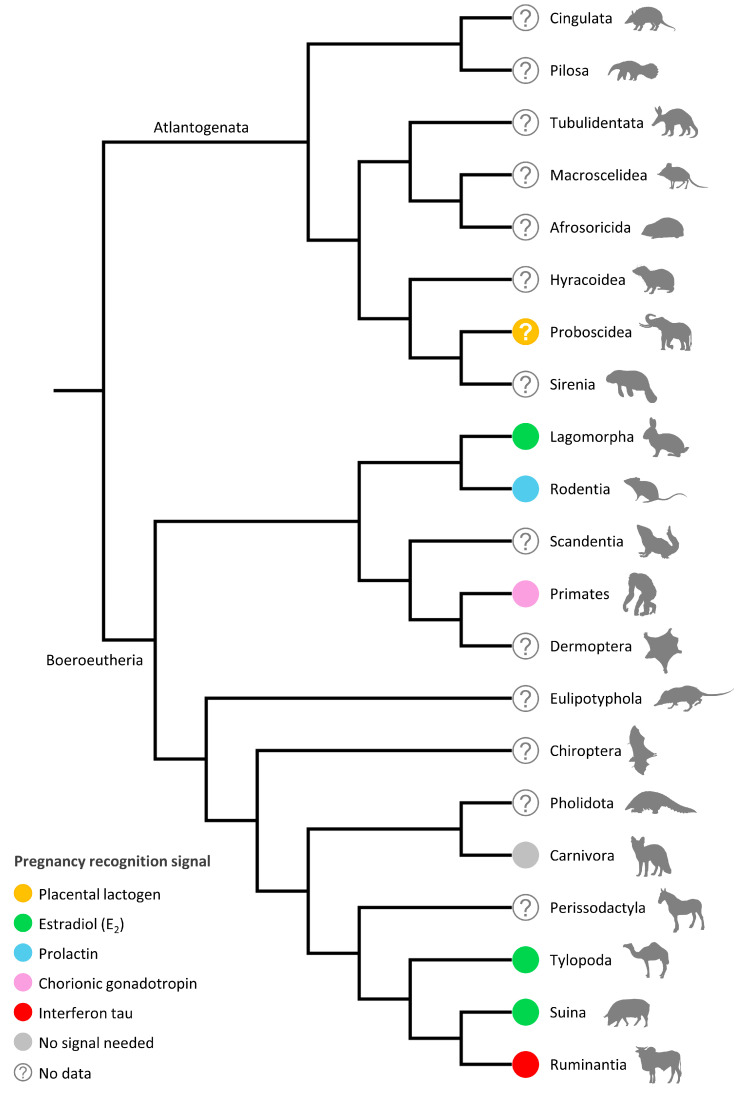
Phylogenetic distribution of the pregnancy recognition signaling in eutherian mammals. Relationships among orders follow Zachos [53].

Phylogenetic and evolutionary patterns of pregnancy recognition signaling remain obscure due to limited data for various lineages (Figure 1). Given the diversity of mammals and pregnancy-related traits, it is likely that there is still a significant amount of variation in pregnancy recognition mechanisms yet to be discovered. Nevertheless, available evidence indicates that mammals have evolved diverse mechanisms to prevent luteolysis. This functional diversity may reflect species-specific adaptations to establish pregnancy.

Artiodactyls are the most extensively studied group regarding maternal recognition of pregnancy. E_2_ is the pregnancy recognition signal in the suborders Tylopoda and Suina, which suggests that it was the ancestral condition in artiodactyls (Figure 1). This hypothesis is supported by the fact that IFN-τ is exclusive to pecoran ruminants. Although other types of interferons are secreted by embryos of other mammalian species (e.g., IFN-α, IFN-β), IFN-τ is absent in all of them, including artiodactyls, such as Suina, Tylopoda, and Cetancodonta [49]. Additionally, the fact that E_2_ also acts as the embryonic signal in lagomorphs implies that its role in pregnancy recognition signaling evolved early in the origin of Boreoeutheria (Figure 1). Gene duplication may also be an important mechanism driving shifts in pregnancy recognition signaling. For example, the CGβ gene, which codes for one of the two chorionic gonadotropin subunits, evolved in the ancestor of anthropoid primates through the duplication of the luteinizing hormone β subunit gene (LHβ), which is widespread among mammals [50]. Moreover, IFN-τ evolved through the duplication of an IFN-ω gene [51]. Gene duplication is an important mechanism for generating phenotypic variation [52]. Further studies are needed to capture the diversity of signaling mechanisms for maternal recognition of pregnancy.

## 4. Implantation

### 4.1. Mechanisms and Modes of Implantation

Implantation is a stepwise process that can be subdivided into apposition, adhesion, and invasion. Apposition initiates when the blastocyst trophectoderm comes into contact with the uterine luminal epithelium. This initial contact involves a close but unstable association between the blastocyst and the uterine luminal epithelium. Next, the apical surface of the blastocyst trophectoderm firmly adheres to the apical surface of the uterine epithelium (and the superficial glandular epithelium in ruminants), establishing a stable fetal-maternal interface [54]. Adhesion is facilitated by the development of interdigitating microvilli between the trophectoderm and uterine epithelium [3]. Apposition and adhesion are steps common to all eutherian mammals and generally occur in a similar manner [3]. In addition, cell-cell interactions and gene expression associated with apposition and adhesion appear quite similar among mammalian species [3,55]. Some eutherian species go a step further, and the blastocyst passes through the endometrium epithelium, invading the basal lamina and stromal vasculature, although the extent of invasion varies among species [3]. Regardless of whether implantation ends at the adhesion or invasion stage, completion of implantation marks the transition to the progressive formation of the placenta that will support development until term.

Implantation can be characterized by the extent to which the blastocyst penetrates the endometrium [14,56,57]. Superficial implantation (sometimes called central implantation) occurs when the blastocyst attaches to and fuses with the uterine luminal epithelium without penetrating it. Interstitial implantation occurs when the blastocyst is fully embedded within the uterine wall. Interstitial implantation takes two forms. The first is secondary interstitial implantation, which occurs when the blastocyst attaches eccentrically in an incubation chamber (or crypt) and then becomes isolated from the uterine lumen through invagination and closure of the uterine epithelium. The second form, primary interstitial implantation, occurs when the blastocyst fully embeds into the uterine endometrium, with subsequent replacement of the epithelium over the invasion site. In some eutherians, the blastocyst partially embeds into the endometrium, a mode referred to as partially interstitial implantation. Therefore, implantation can be achieved with no, minimal, or full invasion of the blastocyst into the endometrium (for illustrations of the different implantation modes, see Figure 5 in Wimsatt [14] and Figure 9 in Luckett [57]).

### 4.2. Patterns of Evolutionary Change in Implantation Modes

Implantation modes have been determined in at least 172 eutherian species (Table 1). To the best of our knowledge, the only order in which the implantation pattern has not been studied is Sirenia. For all other orders, at least one species has been studied. Superficial implantation is widespread in Eutheria, characterizing all representatives in 11 out of 19 eutherian orders (Table 1). One order, Macroscelidea, exhibits only secondary interstitial implantation (Table 1). Variation in implantation patterns has been identified in only four out of the 19 eutherian orders: Primates, Eulipotyphla, Chiroptera, and Rodentia (Table 1). However, even in these orders, superficial implantation is relatively common, characterizing many families (Table 1).

**Figure 2:**
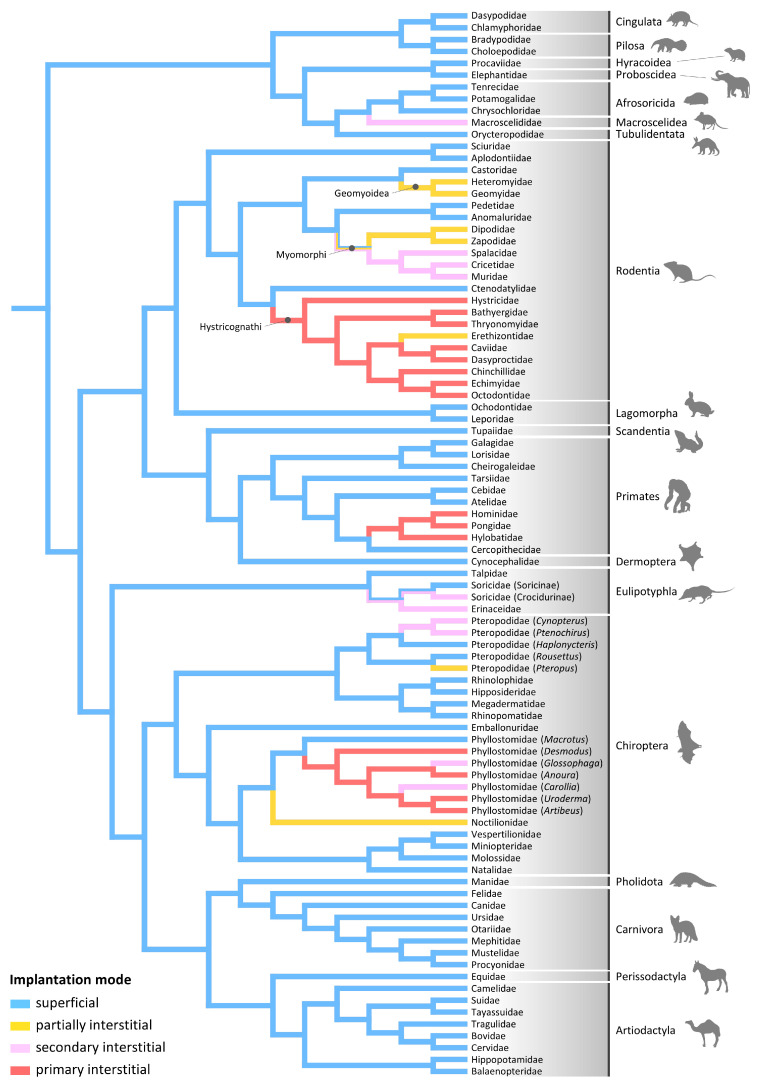
Parsimony reconstruction of the evolution of implantation types in a family-level phylogeny of eutherian mammals. Relationships among orders follow Zachos [53]. Relationships within chiropterans follow Hao et al. [88], Almeida et al. [89], and Camacho et al. [90]. Relationships within rodents follow D’Elía et al. [91] and Blanga-Kanfi et al. [92].

Evolutionary reconstructions suggest that superficial implantation was the ancestral mode of Eutheria (including all its orders and most of its families) and that multiple evolutionary changes in implantation modes have occurred independently (Figure 2; see also [17,67,69,79,80,81]). Partially interstitial implantation has evolved five times in eutherians (Figure 2), with three of these origins occurring within rodents: in the ancestor of Geomyoidea, within Myomorphi (although the node of this transition is unclear), and in the erethizontid *Erethizon dorsatum* (Figure 2). The evolution of partially interstitial implantation in Erethizontidae is intriguing because this family is nested within the infraorder Hystricognathi, a clade composed of species exhibiting primary interstitial implantation (Figure 2). Indeed, primary interstitial implantation has been considered a diagnostic feature of hystricognath rodents (e.g., [75,78]). However, the existence of partially interstitial implantation in *Erethizon dorsatum* was suggested based on the observation of a single early implanting blastocyst [68], and confirmation may be needed [57]. Furthermore, all other aspects of placentation in Erethizontidae closely resemble those of other caviomorphs, and the same may be the case for implantation [57]. The other two origins of partially interstitial implantation in eutherians occurred within chiropterans. The first origin occurred in the genus *Pteropus* (Pteropodidae) and the second in Noctilionidae (Figure 2).

Secondary interstitial implantation has evolved six to seven times in eutherians (Figure 2). One origin occurred in the ancestor of Macroscelidea. One to two origins have occurred in Eulipotyphla. In this order, secondary interstitial implantation may have evolved twice from superficial implantation or once in the ancestor of Erinaceidae + Soricidae, with re-evolution of superficial implantation in the ancestor of Soricinae (Figure 2). Both scenarios are equally parsimonious. Another transition to secondary interstitial implantation occurred within Myomorphi rodents, although the exact node of this transition is ambiguous (Figure 2). Secondary interstitial implantation also arose three times within Chiroptera: once in the ancestor of the pteropodids *Cynopterus* + *Ptenochirus*, and twice within phyllostomids, in *Glossophaga* and *Carollia* (Figure 2). Finally, primary interstitial implantation has evolved three times: once in the ancestor of hystricognath rodents, once within phyllostomid bats, and once in the ancestor of hominoid primates (Figure 2). A potential additional origin of interstitial implantation may have occurred in the galagid *Galagoides demidovii*. This species has been suggested to exhibit interstitial implantation [82], but this claim has been questioned and needs confirmation [54,83,84]. Wimsatt and Enders [85] reported interstitial implantation in the bat *Thyroptera tricolor* (Thyropteridae), but they could not distinguish between primary and secondary types because they lacked earlier implantation stages. Nevertheless, an evolutionary change in implantation type clearly occurred in Thyropteridae.

The distribution pattern of implantation modes in rodents has led several authors to propose that superficial implantation is primitive, whereas primary interstitial implantation is the most derived mode [54,57]. Accordingly, secondary (and presumably partially) interstitial implantation would function as a functionally and evolutionarily intermediate mode of implantation [58,68,77,86,87]. This hypothesis implies that more basal nodes of the phylogeny should exhibit the superficial state, with a progression in subsequent nodes to partially interstitial, secondary interstitial, and finally primary interstitial states. However, phylogenetic reconstructions do not support this idea. Contrary to expectations, all three clades exhibiting primary interstitial implantation evolved this mode from an ancestor exhibiting superficial implantation (Figure 2). Furthermore, among the 6–7 traceable origins of secondary interstitial implantation in eutherians, this mode may have evolved from the partially interstitial mode (i.e., the hypothetical preceding evolutionary state) only in Myomorphi rodents (Figure 2). The evolutionary pattern of implantation modes is more consistent with a scenario in which superficial implantation is ancestral, whereas partial and secondary interstitial modes are distinct derived modes rather than obligatory states between superficial and primary interstitial modes. Regardless of the derived mode of implantation exhibited by a taxon, there is no evidence that reversions to the ancestral mode (superficial implantation) have occurred.

## 5. Concluding Remarks and Future Directions

We provide an overview of the diversity of the processes and the patterns of evolutionary changes in maternal recognition of pregnancy and implantation in eutherians. The different pregnancy recognition signals and implantation modes demonstrate the remarkable diversity of specializations in mammals following the evolution of viviparity. Although significant progress has been made in identifying the mechanisms of pregnancy signaling and implantation modes in various species, much remains to be discovered. Future research should focus on identifying the molecular and genetic underpinnings of pregnancy signals in less-studied clades, such as Chiroptera, Afrotheria, and Xenarthra. Information from a diverse range of taxa will help clarify broader evolutionary patterns of maternal recognition of pregnancy. For example, knowing embryonic signaling in Tragulina (mouse-deers) is key to determining whether IFN-τ is a synapomorphy of ruminants or whether it is specifically of pecoran ruminants.

Superficial implantation is the ancestral implantation mode of Eutheria and its major clades. All other modes (partially, secondary, and primary interstitial) arose independently multiple times in the evolutionary history of eutherians. Ancestral state reconstructions do not support the idea that partially and secondary interstitial implantation are intermediary steps between two extremes (superficial and primary interstitial implantation). However, the implantation mode of various orders is known for only a few members, thus highlighting the need for more comprehensive data. Rodents and chiropterans are particularly important for understanding the transition between implantation modes. To the best of our knowledge, no information is available on the implantation mode of Sminthidae, Platacanthomyidae, Calomyscidae, and Nesomyidae (which belong to Myomorphi). Given that Myomorphi includes species with superficial, partially interstitial, and secondary interstitial implantation, data on these families are essential to determine the direction of evolutionary changes among these modes. Apart from Nesomyidae, these families are not relatively diverse, each containing only one or two genera [93]. Because implantation mode is often phylogenetically conserved at the familial level, studying a few species within these Myomorphi families may be sufficient to determine their implantation mode.

Because variation in the mode of implantation at lower taxonomic levels is rare in eutherians, we emphasize that phyllostomid and pteropodid chiropterans are even more relevant model systems for understanding transitions among implantation modes. Notably, Phyllostomidae is the only eutherian family that exhibits the full range of variation in implantation modes. This diversity provides a valuable opportunity to study the evolutionary transitions between implantation modes within closely related taxa, offering insights that could be extrapolated to broader eutherian patterns. Unfortunately, we currently have a very incomplete picture of how the implantation mode varies in these families. Phyllostomidae (227 species) and Pteropodidae (196 species) are the second and third most diverse bat families, respectively [93]. As more data on their implantation modes become available, a more complex scenario may emerge.

## Figures and Tables

**Table 1:** Mode of implantation in eutherian mammals. Asterisks indicate taxa exhibiting variation in the mode of implantation.

Implantation Type	Order	Family
Superficial	Afrosoricida	Chrysochloridae [54,58,59], Potamogalidae [59], Tenrecidae [54,58,59,60]
	Hyracoidea	Procaviidae [54]
	Proboscidea	Elephantidae [54]
	Tubulidentata	Orycteropodidae [54]
	Cingulata	Chlamyphoridae [54], Dasypodidae [54]
	Pilosa	Bradypodidae [61], Choloepodidae [54]
	Dermoptera	Cynocephalidae [54,58]
	Lagomorpha	Leporidae [54,58], Ochotonidae [54]
	*Primates	Atelidae [58], Cebidae [54,58], Cercopithecidae [58,62], Cheirogaleidae [54], Galagidae [58], Lorisidae [54,58], Tarsiidae [54]
	*Rodentia	Anomaluridae [54], Aplodontiidae [54], Castoridae [54], Ctenodactylidae [54], Pedetidae [54], Sciuridae [54]
	Scandentia	Tupaiidae [58]
	Carnivora	Canidae [54], Felidae [54], Mephitidae [17,63], Mustelidae [54], Otariidae [54], Procyonidae [54], Ursidae [54]
	Artiodactyla	Balaenopteridae [54], Bovidae [54], Camelidae [32], Cervidae [58], Hippopotamidae [64], Suidae [54], Tayassuidae [54], Tragulidae [58]
	*Chiroptera	Emballonuridae [54,56], Miniopteridae [56,65], Molossidae [56], Natalidae [54], Hipposideridae [56], Megadermatidae [56], *Phillostomidae (*Macrotus* [56]), *Pteropodidae (*Haplonycteris* [66], *Rousettus* [54]), Rhinolophidae [56], Rhinopomatidae, Vespertilionidae [56]
	*Eulipotyphla	*Soricidae (Soricinae [54,59,67]), Talpidae [54,59]
	Perissodactyla	Equidae [54]
	Pholidota	Manidae [54]
Partially interstitial	*Chiroptera	*Pteropodidae (*Pteropus* [56]), Noctilionidae [54]
	*Rodentia	Dipodidae, Erethizontidae [54,68], Zapodidae [54]
Secondary interstitial	Macroscelidea	Macroscelididae [69,70,71,72]
	*Chiroptera	*Phyllostomidae (*Carollia* [56], *Glossophaga* [56])
	*Eulipotyphla	Erinaceidae [59], *Soricidae (Crocidurinae [59])
	*Rodentia	Cricetidae [57], Muridae [57], Spalacidae [73]
Primary interstitial	*Chiroptera	*Phyllostomidae (*Anoura* [74], *Artibeus*, *Desmodus* [54,56], *Uroderma* [54])
	*Primates	Hominidae [54], Hylobatidae [54], Pongidae [54]
	*Rodentia	Bathyergidae [75], Caviidae [54], Chinchillidae [54], Dasyproctidae [76], Echimyidae, Hystricidae [75,77], Octodontidae, Thryonomyidae [78]

## References

[1] Blackburn D.G. (2015). Evolution of Vertebrate Viviparity and Specializations for Fetal Nutrition: A Quantitative and Qualitative Analysis. J. Morphol..

[2] Murphy B.F., Thompson M.B. (2011). A Review of the Evolution of Viviparity in Squamate Reptiles: The Past, Present and Future Role of Molecular Biology and Genomics. J. Comp. Physiol. B.

[3] Bazer F.W., Spencer T.E., Johnson G.A., Burghardt R.C., Wu G. (2009). Comparative Aspects of Implantation. Reproduction.

[4] Bazer F.W., Geisert R.D., Fuller F.W. (2015). History of Maternal Recognition of Pregnancy. Regulation of Implantation and Establishment of Pregnancy in Mammals: Tribute to 45 Year Anniversary of Roger V. Short’s “Maternal Recognition Of pregnancy".

[5] Psychoyos A., Greep R.O., Astwood E.G., Geiger S.R. (1973). Endocrine Control of Egg Implantation. Handbook of Physiology.

[6] Bazer F.W., Simmen R.C.M., Simmen F.A. (1991). Comparative Aspects of Conceptus Signals for Maternal Recognition of Pregnancy. Ann. N. Y. Acad. Sci..

[7] Bravo P.W., Diaz D., Alarcón V., Ordoñez C. (2010). Effect of the Reproductive State of Female Alpacas on Embryonic Mortality Rate. Am. J. Vet. Res..

[8] Hyde K.J., Schust D.J. (2015). Genetic Considerations in Recurrent Pregnancy Loss. Cold Spring Harb. Perspect. Med..

[9] Kardong K.V. (2012). Vertebrates: Comparative Anatomy, Function, Evolution.

[10] Seshagiri P.B., Sen Roy S., Sireesha G., Rao R.P. (2009). Cellular and Molecular Regulation of Mammalian Blastocyst Hatching. J. Reprod. Immunol..

[11] Senger P.L. (2005). Pathways to Pregnancy and Parturition.

[12] Paulson E.E., Comizzoli P. (2021). Endometrial Receptivity and Embryo Implantation in Carnivores—Commonalities and Differences with Other Mammalian Species. Biol. Reprod..

[13] Piliszek A., Madeja Z.E., Plusa B., Hadjantonakis A.-K. (2018). Pre-Implantation Development of Domestic Animals. Current Topics in Developmental Biology: Cell Fate in Mammalian Development.

[14] Wimsatt W.A. (1975). Some Comparative Aspects of Implantation. Biol. Reprod..

[15] Renfree M.B., Fenelon J.C. (2017). The Enigma of Embryonic Diapause. Development.

[16] Renfree M.B., Shaw G. (2000). Diapause. Annu. Rev. Physiol..

[17] McGowen M.R., Erez O., Romero R., Wildman D.E. (2014). The Evolution of Embryo Implantation. Int. J. Dev. Biol..

[18] Fenelon J.C., Banerjee A., Murphy B.D. (2014). Embryonic Diapause: Development on Hold. Int. J. Dev. Biol..

[19] Lee K.Y., DeMayo F.J. (2004). Animal Models of Implantation. Reproduction.

[20] Norwitz E.R., Schust D.J., Fisher S.J. (2001). Implantation and the Survival of Early Pregnancy. N. Engl. J. Med..

[21] Green J.A., Geisert R.D., Johnson G.A., Spencer T.E., Geisert R.D., Spencer T.E. (2021). Implantation and Placentation in Ruminants. Placentation in Mammals: Tribute to E. C. Amoroso’s Lifetime Contributions to Viviparity.

[22] Johnson G.A., Bazer F.W., Seo H., Geisert R.D., Spencer T.E. (2021). The Early Stages of Implantation and Placentation in the Pig. Placentation in Mammals: Tribute to E. C. Amoroso’s Lifetime Contributions to Viviparity.

[23] Antczak D.F., Allen W.R., Geisert R.D., Spencer T.E. (2021). Placentation in Equids. Placentation in Mammals: Tribute to E. C. Amoroso’s Lifetime Contributions to Viviparity.

[24] Abdoon A.S., Giraud-Delville C., Kandil O.M., Kerboeuf-Giraud A., Eozénou C., Carvalho A.V., Julian S., Sandra O. (2017). Maternal Recognition of Pregnancy and Implantation Are Not Associated with an Interferon Response of the Endometrium to the Presence of the Conceptus in Dromedary Camel. Theriogenology.

[25] Assis Neto A., Pereira F., Santos T., Ambrosio C., Leiser R., Miglino M. (2010). Morpho-Physical Recording of Bovine Conceptus (*Bos indicus*) and Placenta from Days 20 to 70 of Pregnancy. Reprod. Domest. Anim..

[26] Maddox-Hyttel P., Alexopoulos N., Vajta G., Lewis I., Rogers P., Cann L., Callesen H., Tveden-Nyborg P., Trounson A. (2003). Immunohistochemical and Ultrastructural Characterization of the Initial Post-Hatching Development of Bovine Embryos. Reproduction.

[27] Bazer F.W., Thatcher W.W. (1977). Theory of Maternal Recognition of Pregnancy in Swine Based on Estrogen Controlled Endocrine versus Exocrine Secretion of Prostaglandin F2α by the Uterine Endometrium. Prostaglandins.

[28] Bazer F.W., Johnson G.A. (2014). Pig Blastocyst–Uterine Interactions. Differentiation.

[29] Bazer F.W. (2013). Pregnancy Recognition Signaling Mechanisms in Ruminants and Pigs. J. Anim. Sci. Biotechnol..

[30] Mayor P., Guimarães D.A., López-Béjar M. (2012). Progesterone and Estradiol-17β as a Potential Method for Pregnancy Diagnosis in the Collared Peccary (*Pecari tajacu*). Res. Vet. Sci..

[31] Powell S.A., Smith B.B., Timm K.I., Menino A.R. (2007). Estradiol Production by Preimplantation Blastocysts and Increased Serum Progesterone following Estradiol Treatment in Llamas. Anim. Reprod. Sci..

[32] Bianchi C.P., Gallelli M.F., Herrera J.M., Benavente M.A., Rossetto L., Aba M.A. (2023). Current Knowledge about the Processes of Luteolysis and Maternal Recognition of Pregnancy in Camelids. Reprod. Domest. Anim..

[33] Flint A.P.F., Hearn J.P., Michael A.E. (1990). The Maternal Recognition of Pregnancy in Mammals. J. Zool..

[34] Bazer F.W., Spencer T.E., Norris D.O., Lopez K.L. (2011). Hormones and Pregnancy in Eutherian Mammals. Hormones and Reproduction of Vertebrates, Volume 5—Mammals.

[35] Yamamoto Y., Yamamoto T., Taya K., Watanabe G., Stansfield F.J., Allen W.R. (2011). Placentation in the African Elephant (*Loxodonta africana*). V. The Trophoblast Secretes Placental Lactogen. Placenta.

[36] Lueders I., Niemuller C., Rich P., Gray C., Hermes R., Goeritz F., Hildebrandt T.B. (2012). Gestating for 22 Months: Luteal Development and Pregnancy Maintenance in Elephants. Proc. R. Soc. B Biol. Sci..

[37] Ross G.T., Heap R.B. (1979). Human Chorionic Gonadotropin and Maternal Recognition of Pregnancy. Maternal Recognition of Pregnancy—Ciba Foundation Symposium 64.

[38] Ticconi C., Zicari A., Belmonte A., Realacci M., Rao C.V., Piccione E. (2007). Pregnancy-Promoting Actions of HCG in Human Myometrium and Fetal Membranes. Placenta.

[39] Hearn J.P., Webley G.E., Gidley-Baird A.A. (1991). Chorionic Gonadotrophin and Embryo-Maternal Recognition during the Peri-Implantation Period in Primates. Reproduction.

[40] Thatcher W.W., Binelli M., Burke J., Staples C.R., Ambrose J.D., Coelho S. (1997). Antiluteolytic Signals between the Conceptus and Endometrium. Theriogenology.

[41] Swegen A. (2021). Maternal Recognition of Pregnancy in the Mare: Does It Exist and Why Do We Care?. Reproduction.

[42] Kowalewski M.P., Gram A., Kautz E., Graubner F.R., Geisert R.D., Bazer F.W. (2015). The Dog: Nonconformist, Not Only in Maternal Recognition Signaling. Regulation of Implantation and Establishment of Pregnancy in Mammals: Tribute to 45 Year Anniversary of Roger V. Short’s “Maternal Recognition of Pregnancy”.

[43] Tyndale-Biscoe C.H., Renfree M. (1987). Reproductive Physiology of Marsupials.

[44] Renfree M.B. (2000). Maternal Recognition of Pregnancy in Marsupials. Rev. Reprod..

[45] Renfree M.B. (2010). Marsupials: Placental Mammals with a Difference. Placenta.

[46] Cruz Y.P., Selwood L. (1997). Histological Differences between Gravid and Non-Gravid Uteri in the Dasyurid Marsupial, *Sminthopsis macroura* (Spencer). Reproduction.

[47] Griffith O.W., Chavan A.R., Pavlicev M., Protopapas S., Callahan R., Maziarz J., Wagner G.P. (2019). Endometrial Recognition of Pregnancy Occurs in the Grey Short-Tailed Opossum (*Monodelphis domestica*). Proc. R. Soc. B Biol. Sci..

[48] Freyer C., Zeller U., Renfree M.B. (2003). The Marsupial Placenta: A Phylogenetic Analysis. J. Exp. Zool. A Comp. Exp. Biol..

[49] Carter A.M. (2022). Evolution of Placental Hormones: Implications for Animal Models. Front. Endocrinol..

[50] Maston G.A., Ruvolo M. (2002). Chorionic Gonadotropin Has a Recent Origin within Primates and an Evolutionary History of Selection. Mol. Biol. Evol..

[51] Roberts R.M., Ezashi T., Rosenfeld C.S., Ealy A.D., Kubisch H.M. (2003). Evolution of the Interferon Tau Genes and Their Promoters, and Maternal-Trophoblast Interactions in Control of Their Expression. Reproduction. Suppl..

[52] Magadum S., Banerjee U., Murugan P., Gangapur D., Ravikesavan R. (2013). Gene Duplication as a Major Force in Evolution. J. Genet..

[53] Zachos F.E. (2020). Mammalian Phylogenetics: A Short Overview of Recent Advances. Handbook of the Mammals of Europe.

[54] Mossman H.W. (1987). Vertebrate Fetal Membranes: Comparative Ontogeny and Morphology, Evolution, Phylogenetic Significance, Basic Functions, Research Opportunities.

[55] Spencer T.E., Johnson G.A., Burghardt R.C., Bazer F.W. (2008). Progesterone Regulation of Periimplantation Conceptus Development and Implantation: Genes and Conundrums. Havemeyer Found. Monogr. Ser..

[56] Badwaik N.K., Rasweiler J.J., Crichton E.G., Krutzsch P.H. (2000). Pregnancy. Reproductive Biology of Bats.

[57] Luckett W.P., Luckett W.P., Hartenberger J.L. (1985). Superordinal and Intraordinal Affinities of Rodents: Developmental Evidence from the Dentition and Placentation. Evolutionary Relationships among Rodents.

[58] Mossman H.W. (1937). Comparative Morphogenesis of the Fetal Membranes and Accessory Uterine Structures. Contrib. Embryol. Carnegie Inst. Wash..

[59] Carter A.M., Enders A.C. (2010). Placentation in Mammals Once Grouped as Insectivores. Int. J. Dev. Biol..

[60] Enders A.C., Blankenship T.N., Goodman S.M., Soarimalala V., Carter A.M. (2007). Placental Diversity in Malagasy Tenrecs: Placentation in Shrew Tenrecs (*Microgale* Spp.), the Mole-like Rice Tenrec (*Oryzorictes hova*) and the Web-Footed Tenrec (*Limnogale mergulus*). Placenta.

[61] Heuser C.H., Wislocki G.B. (1935). Early Development of the Sloth (*Bradypus griseus*) and Its Similarity to That of Man. Contrib. Embryol. Carnegie Inst. Wash..

[62] Houston M.L. (1969). The Development of the Baboon (*Papio* Sp.) Placenta during the Fetal Period of Gestation. Am. J. Anat..

[63] Wade-Smith J., Richmond M.E. (1978). Induced Ovulation, Development of the Corpus Luteum, and Tubal Transport in the Striped Skunk (*Mephitis mephitis*). Am. J. Anat..

[64] Amoroso E.C., Hancock N.A., Kellas L. (1958). The Foetal Membranes and Placenta of the Hippopotamus (Hippopotamus Amphibious (Linnaeues)). Proc. Zool. Soc. Lond..

[65] Kimura K., Uchida T.A. (1983). Ultrastructural Observations of Delayed Implantation in the Japanese Long-Fingered Bat, *Miniopterus schreibersii fuliginosus*. Reproduction.

[66] Heideman P.D. (1989). Delayed Development in Fischer’s Pygmy Fruit Bat, *Haplonycteris fischeri*, in the Philippines. Reproduction.

[67] Ferner K., Siniza S., Zeller U. (2014). The Placentation of Eulipotyphla—Reconstructing a Morphotype of the Mammalian Placenta. J. Morphol..

[68] Perrotta C.A. (1959). Fetal Membranes of the Canadian Porcupine, *Erethizon dorsatum*. Am. J. Anat..

[69] Mess A., Carter A.M. (2006). Evolutionary Transformations of Fetal Membrane Characters in Eutheria with Special Reference to Afrotheria. J. Exp. Zool. B Mol. Dev. Evol..

[70] Van der Horst C.J. (1949). The Placentation of *Elephantulus*. Trans. R. Soc. S. Afr..

[71] Oduor-Okelo D., Katema R.M., Carter A.M. (2004). Placenta and Fetal Membranes of the Four-Toed Elephant Shrew, *Petrodromus tetradactylus*. Placenta.

[72] Oduor-Okelo D. (1984). Histology of the Chorioallantoic Placenta of the Golden-Rumped Elephant Shrew (Rhynchocyon Chrysopygus Gunther, 1881). Anat. Anz..

[73] Makori N., Oduor-Okelo D., Otianga-Owiti G. (1991). Morphogenesis of the Foetal Membranes and Placenta of the Root-rat (*Tachyoryctes splendens* (Rüppel)). Afr. J. Ecol..

[74] Hamlett G.W.D. (1935). Notes on the Embryology of a Phyllostomid Bat. Am. J. Anat..

[75] Luckett W.P., Mossman H.W. (1981). Development and Phylogenetic Significance of the Fetal Membranes and Placenta of the African Hystricognathous Rodents *Bathyergus* and *Hystrix*. Am. J. Anat..

[76] Oliveira G.B., Araújo Júnior H.N., Moura C.E.B., Favaron P.O., Pereira A.F., Oliveira M.F. (2023). Placental Development in the Early Stages of Red-Rumped Agouti Pregnancy (*Dasyprocta leporina* Linnaeus, 1758). J. Vet. Sci..

[77] Luckett W.P., Ciochon R.L., Chiarelli A.B. (1980). Monophyletic or Diphyletic Origins of Anthropoidea and Hystricognathi. Evolutionary Biology of the New World Monkeys and Continental Drift.

[78] Oduor-Okelo D., Gombe S. (1991). Development of the Foetal Membranes in the Cane Rat (*Thryonomys swinderianus*): A Re-interpretation. Afr. J. Ecol..

[79] Carter A.M., Mess A. (2008). Evolution of the Placenta and Associated Reproductive Characters in Bats. J. Exp. Zool. B Mol. Dev. Evol..

[80] Luckett W.P., Luckett W.P., Szalay F.S. (1975). Ontogeny of the Fetal Membranes and Placenta: Their Bearing on Primate Phylogeny. Phylogeny of the Primates: A multidisciplinary Approach.

[81] Mess A. (2003). Evolutionary Transformations of Chorioallantoic Placental Characters in Rodentia with Special Reference to Hystricognath Species. J. Exp. Zool. A Comp. Exp. Biol..

[82] Gérard P. (1932). Études Sur l’ovogenèse et l’ontogenèse Chez Les Lemuriens Du Genre *Galago*. Arch. Biol..

[83] Hill J.P. (1965). On the Placentation of *Tupaia*. Proc. Zool. Soc. Lond..

[84] Butler H. (1967). The Giant Cell Trophoblast of the Senegal Galago (*Galago senegalensis senegalensis*) and Its Bearing on the Evolution of the Primate Placenta. J. Zool..

[85] Wimsatt W.A., Enders A.C. (1980). Structure and Morphogenesis of the Uterus, Placenta, and Paraplacental Organs of the Neotropical Disc-winged Bat *Thyroptera Tricolor Spix* (Microchiroptera: Thyropteridae). Am. J. Anat..

[86] Mossman H.W., Hisaw F.L. (1940). The Fetal Membranes of the Pocket Gopher, Illustrating an Intermediate Type of Rodent Membrane Formation. I. From the Unfertilized Tubal Egg to the Beginning of the Allantois. Am. J. Anat..

[87] Keys C.E. (1953). Phyletic Relationships among Some Rodents with Special Consideration of Sigmodon Based on Embryological Data. Trans. Ky. Acad. Sci..

[88] Hao X., Lu Q., Zhao H. (2023). A Molecular Phylogeny for All 21 Families within Chiroptera (Bats). Integr. Zool..

[89] Almeida F.C., Amador L.I., Giannini N.P. (2021). Explosive Radiation at the Origin of Old World Fruit Bats (Chiroptera, Pteropodidae). Org. Divers. Evol..

[90] Camacho M.A., Cadar D., Horváth B., Merino-Viteri A., Murienne J. (2022). Revised Phylogeny from Complete Mitochondrial Genomes of Phyllostomid Bats Resolves Subfamilial Classification. Zool. J. Linn. Soc..

[91] D’Elía G., Fabre P.-H., Lessa E.P. (2019). Rodent Systematics in an Age of Discovery: Recent Advances and Prospects. J. Mammal..

[92] Blanga-Kanfi S., Miranda H., Penn O., Pupko T., DeBry R.W., Huchon D. (2009). Rodent Phylogeny Revised: Analysis of Six Nuclear Genes from All Major Rodent Clades. BMC Evol. Biol..

[93] Mammal Diversity Database (2024). Mammal Diversity Database (Version 1.12.1). https://www.mammaldiversity.org.

